# HollyGTD: an integrated database for holly (Aquifoliaceae) genome and taxonomy

**DOI:** 10.3389/fpls.2023.1220925

**Published:** 2023-07-04

**Authors:** Zhonglong Guo, Junrong Wei, Zhenxiu Xu, Chenxue Lin, Ye Peng, Qi Wang, Dong Wang, Xiaozeng Yang, Ke-Wang Xu

**Affiliations:** ^1^ Co−Innovation Center for Sustainable Forestry in Southern China, College of Biology and the Environment, Nanjing Forestry University, Nanjing, China; ^2^ Institute of Biotechnology, Beijing Academy of Agriculture and Forestry Sciences, Beijing, China; ^3^ WeiRan Biotech, Beijing, China

**Keywords:** holly, Aquifoliaceae, genome, taxonomy, database

## Introduction

Aquifoliaceae, also known as the holly family, comprising the single species-rich genus *Ilex* L. and more than 600 species (Loizeau et al., 2016). Species in this family are dioecious shrubs or trees. It is sub-cosmopolitan, but is best represented in mountainous areas of the tropics, especially in Asia, Central and South America. Many holly species possess great economic value and folk cultural significance. Some of them are commonly used as ornamental plants in parks and gardens for their foliage and decorative berries, such as the common holly *I. aquifolium*, the American holly *I. opaca*, the horned holly *I. cornuta*, and the Japanese holly *I. crenata*. The fruiting branches are also popularly applied to decorate temple courts in China and Christmas trees in the West. Some hollies can also be made into beverages, including *I. paraguariensis* (the “Yerba Mate” or Paraguay Tea in South America), *I. vomitoria* (the “Cassena” or Black Drink in North America and Mexico), *I. latifolia* (Kudingcha in East Asia).

In recent years, genome sequencing has become an important step to decipher the genetic structure and to understand the biological principles controlling the various traits of these plants (Boutanaev et al., 2015; Bredeson et al., 2022; Shen et al., 2023). In order to better store, inquire, mine, integrate, and disseminate the abundant datasets, more and more special comprehensive databases have been launched during the past several years (Harper et al., 2016; Jung et al., 2019; Guo et al., 2023). As a group with important economic value, the genomic and genetic data have been rapidly accumulated for hollies (Kong et al., 2022; Xu et al., 2022a; Yao et al., 2022). However, there is still no integrative database for comparative genomics and transcriptomics of hollies to study gene function and genome evolution. The research community for holly has gathered a significant amount of taxonomic information over the last few decades, including type locality, type specimens, and herbarium code (Manen et al., 2010; Xu et al., 2022b; Yang et al., 2023). But the lack of a standardized platform for data processing and visualization limits the accessibility of such data.

Herein, we developed the Holly Genome and Taxonomy Database (HollyGTD) (https://hollygdb.com/), which integrates the holly data from public databases with the data produced by our group. The HollyGTD combines a variety of multi-omics data (genome, re-sequencing, and transcriptome) and taxonomic resources with a wealth of phenotypic images. HollyGTD offers a couple of easy-to-use access functions/interfaces and eight built-in tools for data analysis, for instance, Blast, JBrowse, Search Gene, Tissue Expression, Gene Annotation, Phylogenetic Tree, Primer Design, and Literature. Therefore, we believe that HollyGTD, a comprehensive database with useful data on genome, genotype, and taxonomy, may represent a valuable resource for the entire holly research community.

## Materials and methods

### Hardware and software

On a Linux server powered by Alibaba Cloud technology, the HollyGTD website is hosted. Technical assistance and web application development have both used the PHP language. The back-end servers were developed by MySQL. HollyGTD’s website interfaces were created using HTML, CSS, and JavaScript. To produce interactive data visualizations, Highcharts (https://www.highcharts.com) was integrated with histograms and heatmaps.

### Resources of genome references and annotations

Two chromosome level genomes in HollyGTD, *Ilex asprella* and *I. polyneura*, were retrieved from NGDC (CNCB-NGDC Members and Partners, 2022) and NCBI (Barrett et al., 2013), respectively. The assembly and annotation of the *Ilex latifolia* genome were done by our group. Genome resources were available in **Supplementary Table S1**.

### Genotyping of re-sequencing data

The raw re-sequencing data of 114 *Ilex* species were produced using Illumina Hiseq X Ten platform by our group (**Supplementary Table S1**). After removing the adapter using trim_galore v0.5.0 (http://www.bioinformatics.babraham.ac.uk/projects/trim_galore/), clean reads were mapped to the *I. latifolia* genome using bwa v0.7.17 (Li, 2013). The variants were then invoked using the standard GATK v4.1.2.0 pipeline (Van der Auwera et al., 2013). SNPs and allele frequency (more than 0.05) were further analyzed. SnpEff v5.1 (Cingolani et al., 2012) was performed to identify SNPs in exons, introns, intergenic regions, 5’ UTRs and 3’ UTRs according the GFF3 file of *I. latifolia*.

### Gene annotation *via* InterProScan

Using InterProScan (5.30), functional domains of protein-coding genes were discovered (Jones et al., 2014). A detailed page with information on homologous, families, domains, repeats, and GO terms was assigned to each gene.

### Taxonomy and phylogenetic tree

Nomenclature of 808 scientific names of Aquifoliaceae were retrieved from Tropicos (https://www.tropicos.org/home) and Jstor (https://www.jstor.org/). Photos of leaves, flowers, pollens, whole plants, and so on were collected from our group. The phylogenetic tree was obtained from Yang’s research (Yang et al., 2023).

### Literature collection

Using the Python Entrez library, automated searches for the terms “*Ilex* AND Aquifoliaceae” were created. Then, 709 holly-related literatures were kept after manual filtration.

### Content of HollyGTD

**Figure 1:**
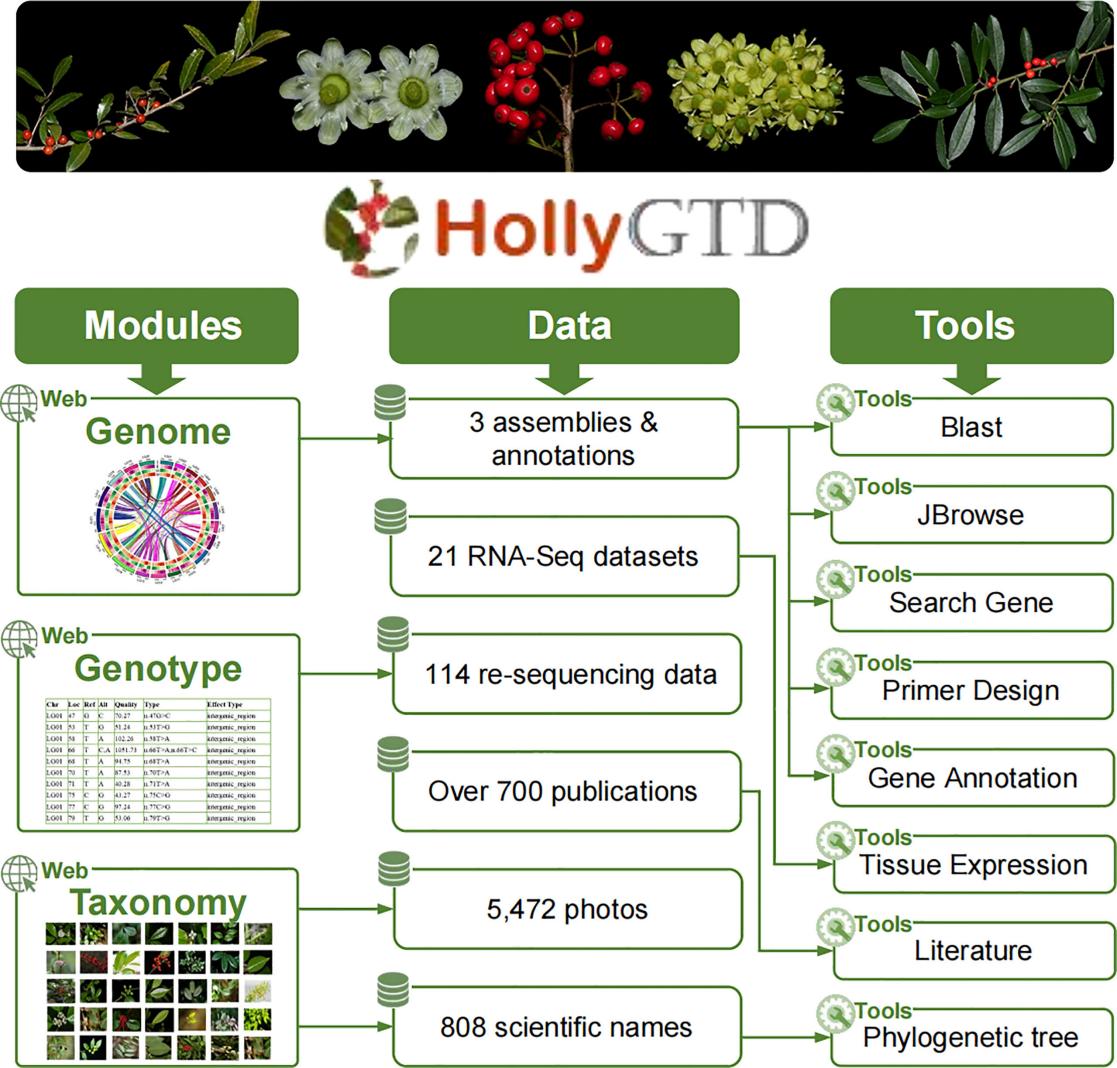
Framework of three parts at Holly Genome Database.

HollyGTD is made up of three parts: modules, data, and tools (**Figure 1**). These three parts work together to better organize all of the current data stored in bulk on HollyGTD and to provide users with user-friendly interfaces and easy-to-use tools.

HollyGTD harbors three major modules or interfaces to present the genome, genotype, and taxonomy datasets (**Figure 1**). Through these modules, users can easily access the underlying data. 1) Genome, which offers comprehensive details on three reference genomes and associated annotations; 2) Genotype, which provides variations produced from re-sequencing data of 114 species *via* visual and searchable access ports; 3) Taxonomy, which houses taxonomic data on every Aquifoliaceae species and arranges all of the manually collected phenotypic images by our group.

Data in HollyGTD include three genomes and associated annotations, 114 re-sequencing data from distinct species of holly, 21 RNA-Seq datasets with different developmental stages, taxonomic information of 808 scientific names, more than 700 research papers published in the last decades, and batched phenotypic photos.

The third part of HollyGTD is designed to create and integrate eight related tools with various functions or data in order to make it easier for users to use and download these data (**Figure 1**). Blast, JBrowse, Primer Design, Search Gene, and Gene Annotation are tools related to various genomics data. Tissue Expression tool interactively displays transcriptomic datasets among distinct developmental stages of fruits and leaves. Phylogenetic Tree enables users to search against the most recent taxonomic relationship of Aquifoliaceae according to Yang’s study (Yang et al., 2023). Literature is used to fast retrieval and access published researches on holly. In addition to these tools, browsers, search engines, filters, and other tools are available to make HollyGTD use easier.

### Tools of HollyGTD

#### Blast

**Figure 2:**
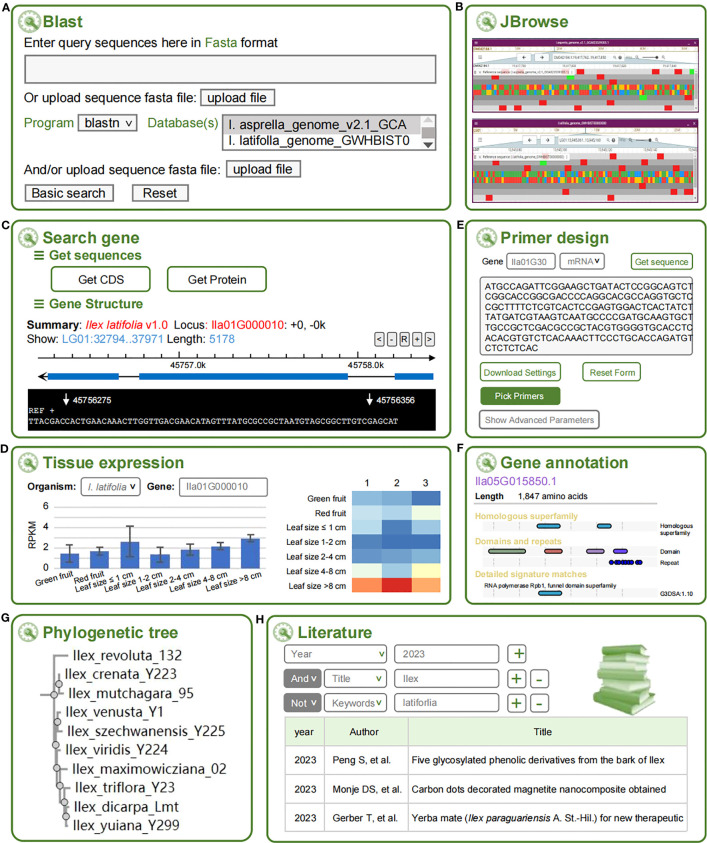
Eight tools at HollyGTD. **(A)** Blast. **(B)** JBrowse. **(C)** Search gene. **(D)** Tissue expression. **(E)** Primer design. **(F)** Gene annotation. **(G)** Phylogenetic tree. **(H)** Literature.

Blast allows users to search the homologous sequences of interest against three holly genomes (**Figure 2A**), either through filling a sequence in the text box or uploading a fasta file. Users can customize their query with advanced options and choose one of the five Blast options (blastn, blastp, blastx, tblastn, or tblastx) that are available. The output results of Blast hits are shown as collapsible fields in a standard table with the following columns: Query name, Target name, Score, Identities, Percentage, and Expect.

#### JBrowse

JBrowse is an open-source, extensible and comprehensive computational platform used to visualize and integrate genomic and multi-omics data (Buels et al., 2016). The integrated data of three genomes and annotated genomic datasets are displayed in HollyGTD using JBrowse2 (**Figure 2B**). HollyGTD currently provides three genome data, and users can easily browse and explore the information they need or are interested in, like the level of expression of particular genes.

#### Search gene

Users can search all annotated holly genes using the Search Gene tool, download the genomics, CDS, and protein of a particular gene, and view the gene structure and sequence using a graphic panel. This tool was developed to make it easier for users to use and download each gene’s information (**Figure 2C**).

#### Tissue expression

Using *I. latifolia* as the reference genome, RNA-Seq datasets were used to determine each gene’s expression level (**Figure 2D**). The Tissue Expression tool can find out the expression level of a given gene in green fruits, red fruits, and different developmental stages of leaves. To visualize the expression data, Highcharts (https://www.highcharts.com) was performed to generate an interactive and dynamic histogram and heatmap. When the cursor is placed over a point on the heatmap, the gene ID, SRR ID, FPKM, and other pertinent data are displayed.

#### Primer design

A web-based PCR primer design tool, Primer-Design, is created with primer3 (Untergasser et al., 2012) as the core program to facilitate the users’ molecular experiment (**Figure 2E**). In addition to the standard primer design function, some novel features for genetic experiment design are available. For instance, by entering the gene ID, the genomic sequences can be automatically loaded into the input field. Additionally, users have a variety of parameters for primer design.

#### Gene annotation

Gene Annotation tool gathers additional functional annotations for each gene, such as detailed information on a specific gene family, homologous superfamily, domains, repeats and GO (Gene Ontology) terms obtained through the InterPro database (Blum et al., 2021) (**Figure 2F**).

#### Phylogenetic tree

Based on the newly generated phylogenetic tree using rigorously identified 202 species and closely authenticated gene sequences of three nuclear genes (ITS, ETS, and *nepGS*), Phylogenetic Tree tool serves users with a convenient web search to retrieve the systematic status of the queried species (**Figure 2G**).

#### Literature

HollyGTD offers a specialized literature retrieval tool for holly scientific research, consisting of more than 700 papers published in the past few decades, to facilitate efficient literature triage and curation (**Figure 2H**). The literature search tool supports keyword searches for years, authors, titles, and journals, while the hyperlinks to full-texts publications are provided in the list of research result.

## Data availability statement

The sources of omics data in HollyGTD are available at **Supplementary Table S1**. The original contributions presented in the study are publicly available. This data can be found here: https://ngdc.cncb.ac.cn/gwh, GWHBIST00000000.

## Author contributions

K-WX, XY and ZG designed the project. ZG and JW designed and developed the HollyGTD website. JW and DW improved the web interface. CL and YP collected and collated the data. ZG and JW performed the bioinformatic analyses. K-WX, ZG and JW wrote the manuscript. All authors contributed to the article and approved the submitted version.
